# Impact of tumor size on subclinical central lymph node metastasis in papillary thyroid microcarcinoma depends on age

**DOI:** 10.1186/s12957-015-0478-9

**Published:** 2015-02-28

**Authors:** Ju-Yeon Kim, Eun Jung Jung, Taejin Park, Sang-Ho Jeong, Chi-Young Jeong, Young-Tae Ju, Young-Joon Lee, Soon-Chan Hong, Sang-Kyung Choi, Woo-Song Ha

**Affiliations:** Department of Surgery, School of Medicine, Gyeongsang National University College of Medicine, 90 Chilamdong, Jinju, 660-702 Korea

**Keywords:** Papillary thyroid microcarcinoma, Lymph node metastasis, Age, Size

## Abstract

**Background:**

The aim of this study is to evaluate whether the associations between clinicopathologic factors of papillary thyroid microcarcinoma (PTMC), especially tumor size, and subclinical central lymph node metastasis (LNM) are dependent on patient age.

**Methods:**

The medical records of 428 patients who underwent thyroid surgery for PTC measuring ≤1 cm were reviewed. All patients were clinically lymph node negative and underwent thyroidectomy with unilateral or bilateral central lymph node dissection. Univariate and multivariate analyses were performed to identify clinicopathologic factors associated with central LNM.

**Results:**

Central LNM was identified in 96 of 428 (22.4%) patients. Mean tumor size was significantly greater in patients with than without central LNM (0.74 ± 0.22 cm vs. 0.64 ± 0.23 cm, *P* = 0.001). Tumor size > 0.5 cm was significantly predictive of central LNM. Subgroup analysis according to age groups showed that tumor size was an independent predictor of subclinical central LNM only in patients aged ≥45 years.

**Conclusions:**

Factors predictive of central LNM in patients with PTMC differed by age. PTMC size was an independent predictor of subclinical central LNM only in patients aged ≥45 years.

## Background

The incidence of thyroid cancer, especially of small tumors measuring ≤1 cm, has been rapidly increasing, due primarily to advances in ultrasonography and fine needle aspiration cytology of the thyroid [1,2]. Although most papillary thyroid microcarcinomas (PTMCs) have an indolent course and excellent prognosis, the long-term recurrence rate of PTMC has been reported to be as high as 12% [3-7], with lymph node metastasis (LNM) being one of the most important factors associated with local recurrence and distant metastasis [6,8,9].

The standard care for patients with thyroid cancer and involved lymph nodes is total thyroidectomy plus therapeutic cervical lymph node dissection. However, it has not yet been determined whether routine central lymph node dissection should be performed in patients with PTMC but without evident LNM. To date, no randomized controlled trials have found that prophylactic central lymph node dissection has prognostic value [10,11]. Although preoperative ultrasonography is commonly recommended in staging of diseases of the thyroid and cervical lymph nodes, this method has limitations in diagnosing central LNM. Its sensitivity and specificity are lower within the central compartment (27.3% to 55% and 69% to 90.3%, respectively) than within the lateral compartment (65% to 90.3% and 82% to 94.8%) [12-15]. Thus, several studies have investigated the preoperative clinicopathologic feature of PTMC that are predictive of central LNM.

Although age at diagnosis is an important prognostic factor in patients with conventional papillary thyroid carcinoma (PTC), it is not prognostic in patients with PTMC; rather, patients with PTMC have different clinical features and prognostic factors according to age [16]. This study analyzed the clinical factors predictive of central LNM according to age in PTMC patients without definite central LNM, with an emphasis on determining whether tumor size is associated with central LNM.

## Methods

PTMC was defined as a subset of papillary thyroid cancer measuring ≤10 mm and has not grown outside the thyroid (pT1a) by American Joint Committee on Cancer (AJCC)/World Health Organization (WHO) 2009 tumor node metastasis (TNM). Before this definition, the only criterion of PTMC was the size regardless of having extrathyroidal extension of tumor. So we analyzed all PTC measuring ≤10 mm, and the subgroup of tumor without extrathyroidal extension (true PTMC) was subanalysed. Tumor size was confirmed by surgical pathology in all patients. Preoperative physical examination and ultrasonography showed that all patients were clinically lymph node negative.

The medical records of patients who underwent thyroid surgery for malignancy from January 2005 to December 2013 in our hospital were reviewed. Patients with other malignancies and those with a history of previous thyroid surgery were excluded. Of the 1,058 patients with PTC, 538 patients were diagnosed as having a tumor of size 10 mm. Among them, 428 patients underwent thyroidectomy with unilateral or bilateral central lymph node dissection.

Patients were divided into two groups by age at diagnosis, <45 and ≥45 years, respectively. Tumor sizes in patients with and without LNM were compared using Student’s *t*-tests. Univariate and multivariate analyses were performed to identify clinicopathologic factors associated with central LNMs. Odds ratio (OR) and 95% confidence interval (CI) were calculated to determine the relevance of all potential predictors of central LNM. Cumulative central LNM risk was determined using the Kaplan-Meier method and compared using the log-rank test. All statistical analyses were performed using SPSS (version 19.0 SPSS Inc., Chicago, IL, USA). Significance was defined as *P* < 0.05. The study protocol including the use of the database was approved by the Institutional Review Board of Gyeongsang National University Hospital and met the guidelines of the responsible governmental agencies.

## Results

The clinical and histopathological characteristics are given in Table 1. Of the 428 patients, they ranged in age from 15 to 80 years, with 264 patients being ≥45 years old. Central LNMs were identified in 96 patients (22.4%), with 25 (5.8%) having four or more LNMs. By definition, 282 patients were classified as PTMC.

**Table 1:** **The clinical and histopathological characteristics of the patients with papillary thyroid cancer measuring ≤1 cm**

**Characteristics**	**All patients**	**Patients without extrathyroidal extension**
Enrolled patients	428	282
Sex (M/F)	67/361 (15.7%/84.3%)	44/238 (15.6%/84.4%)
Age (years), mean ± SD (range)	48.54 ± 12.31 (15 to 80)	48.27 ± 12.33 (15 to 77)
<45	164 (38.3%)	108 (38.3%)
≥45	264 (61.7%)	174 (61.7%)
Extent of thyroidectomy		
Total thyroidectomy	297	185 (65.6%)
Less than total	131	97 (34.4%)
Tumor size (cm), mean ± SD (range)	0.66 ± 0.23 (0.1 to 1.0)	0.67 ± 0.23 (0.1 to 1.0)
Multifocality (%)	116 (27.1%)	67 (23.8%)
Central lymph node metastasis (%)	96 (22.4%)	52 (18.4%)
No. of metastasis		
1 to 3	71	37
4 to 10	25	15
Thyroiditis (%)	53 (12.4%)	46 (16.3%)
Extrathyroidal extension (%)	146 (34.1%)	

M, male; F, female; SD, standard deviation; No., number.

### Factors associated with central LNM of patients with PTC measuring ≤1 cm

Univariate analysis showed that central LNM of patients with PTC measuring ≤1 cm was significantly associated with tumor size, multifocality, and extrathyroidal extension. The rate of central LNM was higher in patients <45 years than that in patients ≥45 years (26.8% vs. 19.3%, *P* = 0.068) However, patient age was not significantly associated with LNM using cutoffs of 40, 45, and 50 years. Multiple logistic regression analysis showed that tumor size >0.5 cm was an independent predictor of central LNMs in study patients (OR = 1.967 *P* = 0.015) (Table 2).

**Table 2:** **Analysis for risk factors of central lymph node metastasis of the patients with papillary thyroid cancer measuring ≤1 cm**

	**Univariate analysis**			**Multivariate analysis**		
	**Odd ratio**	***P*** **value**	**95% CI**	**Odd ratio**	***P*** **value**	**95% CI**
Sex (male)	0.719	0.425	0.37 to 1.41			
Age ≥ 45 years	0.378	0.057	0.40 to 1.00			
Size > 0.5 cm	2.084	0.006	1.23 to 3.52	1.967	0.015	1.14 to 3.39
Mutifocality	2.554	<0.001	1.58 to 4.13	2.269	0.001	1.39 to 3.70
Extrathyroidal extension	1.908	0.006	1.20 to 3.04	1.699	0.030	1.05 to 2.74
Thyroiditis	1.143	0.696	0.58 to 2.24			
Preoperative TSH (continuous variable)	0.917	0.307	0.78 to 1.08			

A multivariate analysis was set by using all of the predictors with p values under 0.05 in univariate analysis.

TSH, thyroid stimulating hormone; CI, confidence interval.

### Tumor size as a predictor of central LMNs according to age group

**Figure 1:**
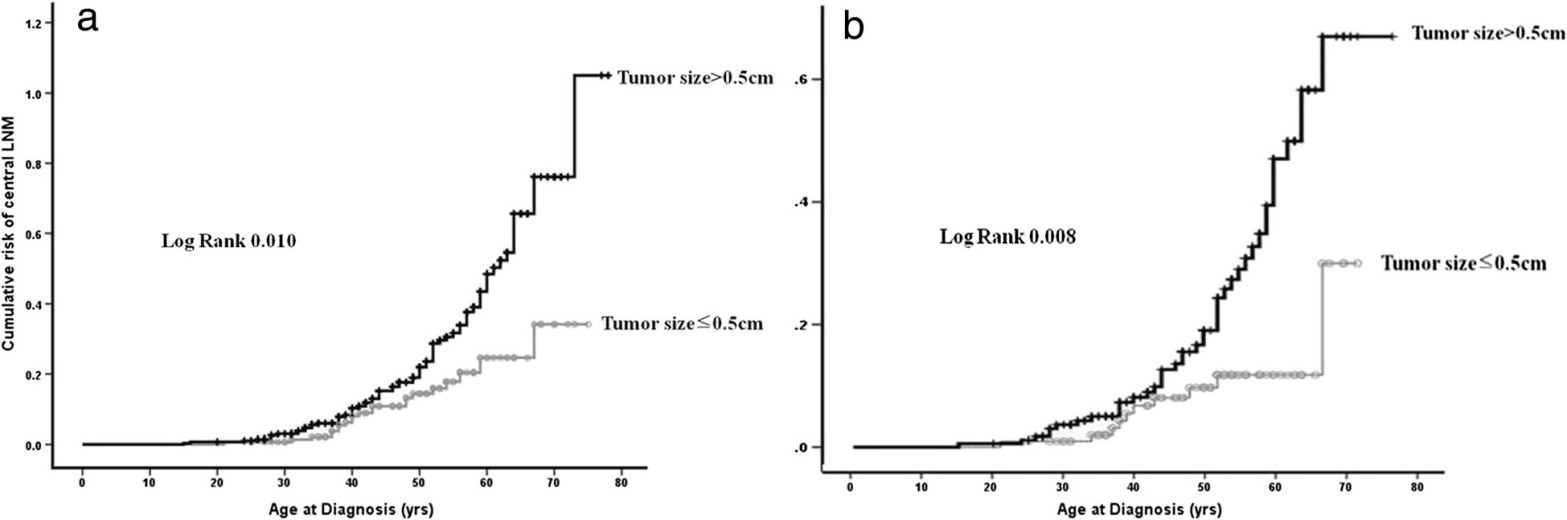
**Cumulative risk of central lymph node metastasis according to age at diagnosis.** **(a)** All PTC patients with tumor measuring <1 cm. **(b)** PTMC patients.

Mean tumor size was significantly larger in patients with than without central LNM (0.74 ± 0.22 vs. 0.64 ± 0.23 cm, *P* = 0.001). Among patients <45 years old at the time of diagnosis, mean tumor size did not differ by LNM status (*P* = 0.635). However, in patients aged ≥45 years at diagnosis, tumor size was significantly larger in patients with than without LNM (0.71 vs. 0.61 cm, *p* = 0.018). We therefore analyzed the cumulative risk of central LNM in each age group, by comparing patients with tumors <0.5 and ≥0.5 cm. Among patients <45 years old, those with PTMCs <0.5 and ≥0.5 cm had similar cumulative risks of central LNM. However, among patients ≥45 years old, those with larger tumors had a greater cumulative risk of central LNM than those with smaller tumors (Figure 1).

Logistic regression analysis was performed to determine factors significantly associated with central LNM in the two age groups. In patients ≥45 years old, large tumor size (OR = 3.167, *P* = 0.006) and multifocality (OR = 2.098, *P* = 0.025) were significantly associated with central LNM. In patients <45 years old, multifocality was the only significant predictor of central LNM (OR = 2.829, *P* = 0.009); tumor size was not associated with central LNM in this group (*P* = 0.635) (Table 3).

**Table 3:** **Risk factors of central lymph node metastasis according to age groups in all patients**

	**Group I (age < 45 years)**			**Group II (age ≥ 45 years)**		
	**Odd ratio**	***P*** **value**	**95% CI**	**Odd ratio**	***P*** **value**	**95% CI**
Size > 0.5 cm	1.206	0.635	0.56 to 2.62	3.167	0.006	1.40 to 7.16
Multifocality	2.829	0.009	1.30 to 6.18	2.098	0.025	1.10 to 4.02
Extrathyroidal extension	1.935	0.075	0.94 to 3.99	1.534	0.194	0.80 to 2.93

### Factors associated with central LNM of patients with PTMC

The result was similar in patients with PTMC (without extrathyroidal extension). The rate of central LNM was higher in patients <45 years than that in patients ≥45 years (22.2% vs. 16.1%). Tumor size was the only significant predictor of central LNM in PTMC patients ≥45 years old (OR = 6.875, *P* = 0.002) (Table 4).

**Table 4:** **Risk factors of central lymph node metastasis according to age groups in patients without extrathyroidal extension**

	**Group I (age < 45 years)**			**Group II (age ≥ 45 years)**		
	**Odd ratio**	***P*** **value**	**95% CI**	**Odd ratio**	***P*** **value**	**95% CI**
Size > 0.5 cm	1.420	0.485	0.53 to 3.80	6.875	0.002	1.99 to 23.78
Multifocality	2.731	0.057	0.97 to 7.68	2.052	0.097	0.88 to 4.79
Sex (male)	0.282	0.553	0.32 to 3.72	0.223	0.082	0.02 to 1.38
Thyroiditis	0.497	0.380	0.10 to 2.37	1.694	0.282	0.65 to 4.43
TSH	0.900	0.582	0.62 to 1.31	0.945	0.678	0.73 to 1.23

TSH, thyroid stimulating hormone; CI, confidence interval.

## Discussion

Results from this study showed that the size of PTMCs was an independent predictor of subclinical central LNM only in patients ≥45 years old at the time of diagnosis. Although other studies assessed the clinicopathological factors associated with subclinical central LNM in patients with PTMC, those results were inconsistent. In particular, it was unclear whether tumor size and age at diagnosis were predictors of subclinical central LNM.

Previous studies have suggested that tumor size is an independent predictor of subclinical central LNM of PTMC [17-23], but the size cutoffs differed across those studies. Although a study reported that a threshold of 6 mm may be more appropriate than 5 mm [23], most studies to date have used 5 mm as the size threshold and have analyzed the aggressiveness of PTMCs. In the study by Parkdaman *et al*. [19], there were no differences in lymph node metastasis according to cutoff of size 5 vs. 4 mm. Also, in another study of multivariate analysis [24], they found that a size cutoff of 5 mm was not an independent predictor of subclinical central LNM. In our study, the analysis of all patients showed that tumor size, both as a continuous variable and using a cutoff of 5 mm, was a significant predictor of central LNM. However, in the subgroup of PTMC patients <45 years at diagnosis, tumor size was not associated with central LNM.

**Figure 2:**
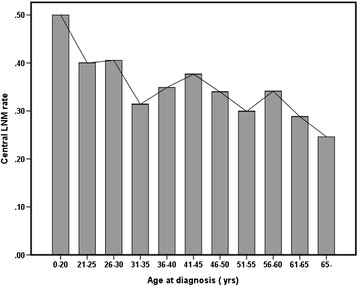
**Central lymph node metastasis rate in patients aged <45 and ≥45 years.**

Age is an important prognostic factor in patients with PTCs > 1 cm; however, its prognostic value in PTMC has been uncertain, with inconsistent results among studies [17,20,23-25]. A cutoff age of 45 years is widely used as a clinically prognostic marker [22]. In analyzing the incidence of central LNM according to patient age, we observed a trend to an inverse relationship. However, a detailed analysis showed fluctuations according to age groups (Figure 2). Hence, the difference in age distribution in each of the previous studies doubtlessly affected the associations between age and central LNM.

This study found that multifocality was a significant predictor of central LNM regardless of age group in patients with PTC measuring ≤1 cm. Similarly, a meta-analysis of 1,789 patients with PTMC showed a significant association between multifocality and LNM in PTMC (OR, 1.9; 95% CI 1.6 to 2.4, *P* < 0.001) [25].

This study confirmed that factors predictive of central LNM in patients with PTMC differed by age. It is important to determine whether central LNM is associated with prognosis in PTMC patients. Although lateral neck LNM has been considered prognostic in patients with PTMC, the prognostic importance of central LNM is unclear. Several studies found that central LNM was not prognostic [6,10]. However, we previously reported that the patients with PTMCs had different clinical features and prognostic factors according to age [16]. Thus, regardless of the relationship of central LNM and prognosis, clinicians who treat patients with PTMC should tailor treatment and follow-up to patient age.

This study has several limitations. Due to its retrospective nature, there may have been a selection bias. Moreover, our study population was a cohort of patients cared for in a single center. The limited number of our patients, despite the statistical adjustments for small specimens, compels us to emphasize the need for further studies, with larger series of patients, in order to test our results. Also, we only focused the clinicopathologic factors to predict central LNM irrespectively of the imaging features of PTMC such as ultrasound or computer tomography.

## Conclusions

The rate of central LNM in PTMC patients ≥45 years old was significantly low in patients who had small tumor measuring ≤0.5 cm. Therefore, these patients might be safe without prophylactic central LN dissection. However, tumor size in PTMC patients <45 years old was not a predictive factor of central LNM.

## Abbreviations

LNM: lymph node metastasis
OR: odds ratio
PTC: papillary thyroid carcinoma
PTMC: papillary thyroid microcarcinoma
